# Telemonitoring of Children with COVID-19: Experience Report of the First 100 Cases

**DOI:** 10.1089/tmr.2020.0006

**Published:** 2021-01-22

**Authors:** Thales Araújo de Oliveira, Ary Costa Ribeiro, Felipe Monti Lora, Francisco Ivanildo de Oliveira, Rogério Carballo Afonso

**Affiliations:** ^1^ Department of Emergency, Sabará Hospital Infantil, São Paulo, Brazil.; ^2^ Department of Superintendence, Sabará Hospital Infantil, São Paulo, Brazil.; ^3^ Department of Quality, Sabará Hospital Infantil, São Paulo, Brazil.; ^4^ Department of New Technology, Sabará Hospital Infantil, São Paulo, Brazil.

**Keywords:** COVID-19, children, telemedicine, telemonitoring

## Abstract

**Introduction:**

The first case of coronavirus disease 2019 (COVID-19) in Brazil was diagnosed in February 2020. On March 20, the Ministry of Health issued Ordinance no. 467, regulating the use of telemedicine during the pandemic period. One of the various modalities of telemedicine is telemonitoring.

**Objective:**

To report our experience with telemonitoring and evaluate its applicability in the follow-up of the first 100 children who received the diagnosis of COVID-19 after visiting the emergency department of Sabará Hospital Infantil (“Hospital Sabará”) and who had no indications for hospitalization.

**Methods:**

The care records of the children were retrospectively analyzed, and telephone contact with the families of patients who did not complete the proposed telemonitoring protocol was initiated.

**Results:**

The average age of the children was 5.5 years, and a slight male predominance (54/100) was observed. Comorbidities were present in 24/100. The source of infection was family members living in the same household in 88/100 and other sources in 12/100. In the first telemonitoring, 44% of the evaluated patients were asymptomatic. In the second telemonitoring, 81% of the patients were asymptomatic. Telemonitoring was completed by 70% of the children. A total of 14 children returned to the emergency department, 11 of whom spontaneously (2/11 were admitted) and 3 under the indication of telemedicine (3/3 were admitted).

**Conclusions:**

Telemonitoring proved to be a clinically valuable resource in the follow-up of children with COVID-19, as it allowed continuity of care and identified patients with indications to return to the emergency department of Hospital Sabará and for hospitalization, thus avoiding unnecessary emergency department visits.

## Introduction

The first case of coronavirus disease 2019 (COVID-19) in Brazil was diagnosed in February 2020.^1^ On March 11, 2020, the World Health Organization officially declared the global pandemic status of COVID-19. As a result, numerous international borders were closed and social isolation became the recommended and adopted measure not only in Brazil but also worlwide.^2^

In this context, on March 20, 2020, the Brazilian Ministry of Health issued Ordinance no. 467, regulating the use of telemedicine during the pandemic period.^3^ The COVID-19 pandemic stimulated significant changes in health care, such as accelerating the adoption of telemedicine.^4^

According to Federal Medical Council of Brazil (CFM) Resolution no. 1643/2002, telemedicine is defined “as the exercise of medicine through the use of interactive audiovisual communication and data methodologies, with the objective of health care, education, and research”.^5^

The incorporation of new technologies in the hospital environment has been increasingly realized in recent years, including the use of artificial intelligence, robots, and telemedicine itself. These new technologies have shown promising utility in the prevention and monitoring of diseases, and have especially contributed to increasing the level of treatment adherence among children and adolescents.^6^ For instance, studies have shown the potential of using mobile phones to help patients with diabetes in effectively managing their condition.^7^

Remote patient care has been considered essential in the provision of health services worldwide during the COVID-19 pandemic.^8^ In this context, telemonitoring, which is defined as the provision of medical guidance through the remote monitoring of a patient's health or disease parameters, is an important modality.^9^

Telemonitoring is an important tool that enables the continuity of health care provision, to the extent of identifying possible treatment complications, especially for patients who wish to maintain social distancing and avoid unnecessary visits to the hospital or clinic.^10^ This medical service modality has a crucial role in minimizing physical contact between doctors and patients, thus contributing to breaking the chain of infection and optimizing health services.^11^

Indeed, with the implementation of the telemedicine service in the emergency department of Sabará Hospital Infantil (“Hospital Sabará”), telemonitoring of patients diagnosed with COVID-19 has proved to be relevant for the continuity of care at a time when social isolation is prioritized (according to guidelines from health authorities to avoid visiting emergency departments and based on the fear of families related to face-to-face medical evaluations).

This article aimed to report our experience with telemonitoring in the first 100 children diagnosed with COVID-19 in the emergency department of Hospital Sabará, and to describe the demographic and epidemiological characteristics of these patients. We also evaluated the usefulness of telemedicine in the follow-up of children with COVID-19 in relation to the detection of clinical worsening and the determination of the need for hospitalization or in-person re-evaluation.

We hypothesized that the use of telemedicine can help in the fight against the COVID-19 pandemic.

## Methods

Hospital Sabará is the second largest private and exclusively pediatric hospital in Brazil. Its emergency department receives an average of 100,000 children annually. It has 112 inpatient beds, distributed in inpatient units (70 beds) and intensive care units (42 beds).^12^

This was a retrospective observational study that analyzed the medical records of patients cared for in the emergency department of Hospital Sabará from April 17 to July 2, 2020, and were diagnosed with COVID-19. The data of the participating children and their family members were anonymized. Therefore, this experience report reveals no data of an identified or identifiable person and ensures the right of privacy of the analyzed patients, in accordance with the ethical principles of medicine. This study was approved by the research ethics committee of Fundação José Luiz Egydio Setubal (CAAE: 36495720.4.0000.5567).

A total of 100 children were studied, and follow-up was performed using telemedicine. After a series of 100 cases, telephone contact was made with the families of the children who did not complete the telemonitoring protocol, to identify the causes of noncompletion and obtain data on the clinical outcome.

To perform telemonitoring in the emergency department, we employed the telemedicine platform licensed to Hospital Sabará by Conexa Saúde^®^, which uses the Conexa network and the White Label platform (licensed software). After the appointment, a link to register on the platform was sent to the guardians. During the registration, free and informed consent terms were presented about the use of the technological platform for providing the service and about the personal data that will be processed by the hospital.

This study included children aged from 0 to 17 years with a confirmed diagnosis of COVID-19, who were sent home after visiting the emergency department and signed the free and informed consent form for the use of telemedicine as medical care. Patients who needed the telemedicine service for other pathologies or those diagnosed with COVID-19 with indications for hospitalization were excluded from the analysis.

All patients received initial in-person care following the institutional protocol. After the screening process, the pediatrician identified whether the child had, in the past 14 days, respiratory symptoms of probable infectious cause (with or without fever), such as cough, nasal congestion, runny nose, sore throat, dyspnea, or positive epidemiological history of exposure to a COVID-19 patient. If any of these symptoms were present, the patient was included in the group of suspicious children who subsequently underwent nasopharyngeal and oropharyngeal swabs for severe acute respiratory syndrome coronavirus 2 (SARS-CoV-2) for molecular detection using reverse transcription polymerase chain reaction (RT-PCR).

**FIG. 1.**
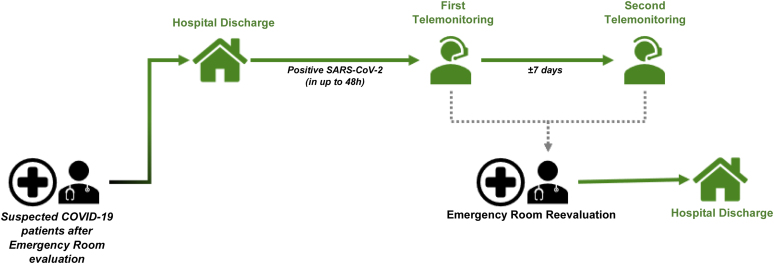
Telemedicine.

Children suspected to have COVID-19 but had no indications for hospitalization were sent home after the collection of samples for RT-PCR for SARS-CoV-2. After the release of the RT-PCR test results by the laboratory, those with a positive result were included in the telemonitoring protocol, which was composed of two steps: (1) “first telemonitoring” performed within 48 h of discharge from the emergency department to inform the patients and their families about the positive result of the test for SARS-CoV-2 and to conduct the first clinical evaluation, and (2) “second telemonitoring” performed 7 days after the first telemonitoring to re-evaluate the patients' clinical condition (Fig. 1).

In both steps, the guardians were asked about the evolution of respiratory symptoms; the presence of fever, vomiting, diarrhea, diuresis, and skin spots; and the food acceptance of the children. The two steps of telemonitoring were performed to follow up the evolution of COVID-19 in the patients, to proactively identify the possible need to return to the emergency department and for hospitalization.

## Results

Between April 17 and July 2, 2020, 1237 patients cared for in the emergency department of Hospital Sabará had a diagnostic suspicion of COVID-19. Among all patients with clinical suspicion (age 0–17 years), 124 (10%) had a diagnosis of COVID-19 confirmed by a nasopharyngeal or oropharyngeal swab test for SARS-CoV-2 by RT-PCR. Of these 124 children, 24 were admitted after face-to-face evaluation in the emergency room (12 in an inpatient unit and 12 in an intensive care unit).

**FIG. 2.**
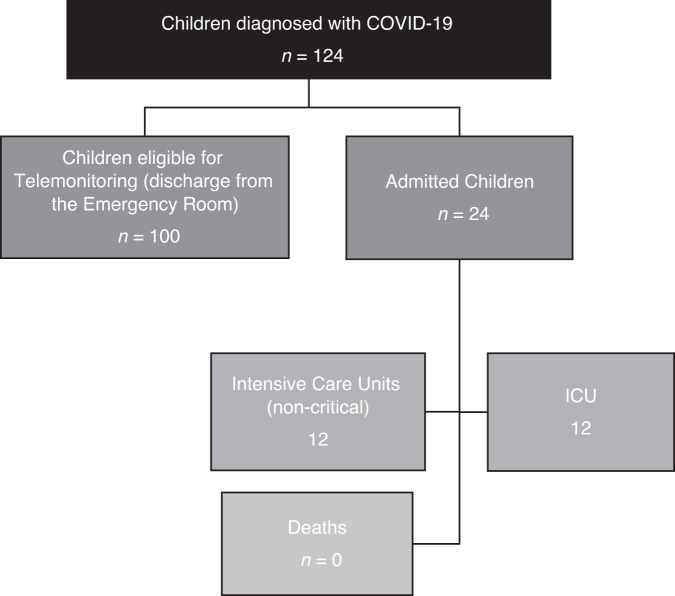
Subpopulation of the 124 children diagnosed with COVID-19 from the Hospital Sabará in the period from April 17th to July 2nd, 2020. COVID-19, coronavirus disease 2019.

The remaining 100 patients who had no indications for hospitalization after the first visit to the emergency department of Hospital Sabará constituted the group of eligible children and were included in the telemonitoring group (“study group”). Figure 2 describes the population of 124 children diagnosed with COVID-19 at Hospital Sabará during this period.

The average age of the children in the study group was 5.5 years, and a slight male predominance (54/100) was observed. The presence of comorbidities was verified in 24 of the 100 children, among which neurological conditions, asthma, and trisomy 21 were the most common. With respect to the infection source, family members living in the same household were determined to be the source in most of the cases (88/100). A different source was identified in only 12 of the 100 children.

The epidemiological profile of the study group was compared with that of patients investigated by Parri et al.^13^ (Table 1).

**Table 1.** Age Distribution of the 100 Children Diagnosed with COVID-19 Compared with the Study

Epidemiological characteristics	Study group (n: 100)	Parri et al.^13^ NEJM, May 1, 2020 (n: 100)
Age—distribution	0–17	0–17.5
Average age	5.5	3.3
<1 year	22	40
1–6 years	39	15
6–10 years	18	21
>10 years	21	24

COVID-19, coronavirus disease 2019.

The first telemonitoring was successfully performed in 92 patients (92%), and the second telemonitoring in 70 patients (70%). The causes of failure in telemonitoring were difficulties in contacting the patients for scheduling purposes (18), unavailability of time of the families (5), insecurity of family members in performing the care of their children with remote guidance (3), internet connectivity issues (2), and decision of some families to continue the care of their children using other health services (2).

After the second telemonitoring, telephone contact was made with 30 families whose children did not fully adhere to the protocol. The contact was made by only one physician who performed structured interviews to evaluate clinical evolution, possible admissions to other institutions, and reasons for nonadherence to the proposed telemonitoring protocol. A maximum of three attempts were made to contact each family. The contact was successful in 28 cases. Successful contact was made in all eight families that did not comply with the protocol of the first telemonitoring. Among the 22 families that did not comply with the protocol of the second telemonitoring, 20 were successfully contacted. Thus, considering the 70 cases that fully adhered to the telemonitoring protocol and the 28 cases with successful telephone interviews, our follow-up rate in this series was 98%.

**FIG. 3.**
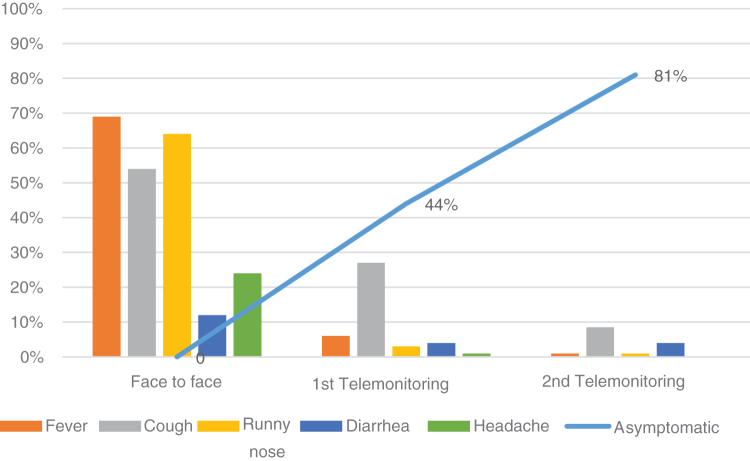
Evolution of symptoms of the children with COVID-19 at the time of face-to-face care (*n* = 100), in the first telemonitoring (*n* = 92) and in the second telemonitoring (*n* = 70).

In the first telemonitoring, performed within 48 h of discharge from the emergency department, 44% (*n* = 92) of the evaluated patients were already asymptomatic. In the second telemonitoring, performed in the subsequent 7 days, the percentage of asymptomatic patients was 81% (*n* = 70). That is, in most of the evaluated cases, there was no persistence of symptoms 9 days after the first visit to the emergency department of Hospital Sabará, as shown in Figure 3.

The symptoms found in the evaluated children are typical of those described in the medical literature related to SARS-CoV-2 infections, considering the limited reports in the pediatric population. Telemonitoring showed a rapid improvement of the condition in the children, similar to other viral conditions affecting this age group.

Clinical worsening was identified in 3 of the 92 patients who underwent the first telemonitoring, and these patients were indicated to return to the emergency department of Hospital Sabará for in-person care. Of the 70 patients who underwent the second telemonitoring, none were indicated to return to the emergency department.

Among the 98 patients in the study group, considering the 70 patients who fully adhered to the telemonitoring protocol and the 28 patients who underwent a subsequent telephone interview, 14 patients returned to an emergency service.

The average age of the children who returned was 4.5 years. Of these, three children had comorbidities. The reasons for the return were tiredness (*n* = 3), persistent fever (*n* = 3), headache (*n* = 2), irritability (*n* = 1), lack of appetite (*n* = 1), need for guidance (*n* = 10), epistaxis (*n* = 1), colic (*n* = 1), and presence of rash (*n* = 1).

Among the 14 children who returned to the emergency department, only 3 returned under the indication of telemedicine. These three patients were indicated for hospitalization (one in the intensive care unit and two in the inpatient unit). In 11 cases, the families spontaneously decided to return to an emergency service without an indication under the telemonitoring protocol. Of them, 10 returned to the emergency department of Hospital Sabará and 1 sought emergency service from another hospital. Of these 11 cases, 2 had indications for hospitalization (1 in Hospital Sabará and 1 in a different hospital, both in inpatient units).

Telemedicine indicated the need to return to the emergency department, as a tool that determines the risk of hospitalization. Although the low number of cases leads to a large uncertainty of the odds ratio (odds ratio [OR], 26.6; 95% confidence interval, 1.006–702.98), the OR achieved statistical significance (*p* = 0.0495), which suggests the clinical utility of telemedicine in reducing unnecessary visits to the emergency department, as shown in Table 2.

**Table 2.** Indicators Considered for the *Odds Ratio* Calculation

Outcome	Telemedicine indication	
	With return indication	Without return indication
Admitted	3	2
Not admitted	0	9

The average length of stay of the patients hospitalized after the first care in the emergency department (*n* = 24) was 5 days and that of patients hospitalized after telemonitoring was 4 days. Although 24 (24%) children in the study group (*n* = 100) presented at least one comorbidity, only 1 child in this group needed to be hospitalized, as shown in Table 3.

**Table 3.** Data from the 14 Children Diagnosed with COVID-19 Who Returned to an Emergency Service for Face-to-Face Evaluation

Presencial emergency room returns	N (%)
Returns to emergency room	14
Average age (years)	4.5
Presence of comorbidity	3 (21)
Average return time	3.2 days
Guided by telemonitoring	3 (22)
On their own	11 (78)
Reason for return	
Tiredness	3 (22)
Persistent fever	3 (22)
Headache	2 (14)
Irritability	1 (7)
Lack of appetite	1 (7)
Guidance	1 (7)
Epistaxis	1 (7)
Colic	1 (7)
Rash	1 (7)
Total hospitalizations	5
Guided by telemonitoring	3/5
On their own	2/11

## Discussion

Although a literature review has already demonstrated how telemonitoring plays a crucial role in minimizing physical contact between doctors and patients, optimizing the supply of health services, and contributing to social distancing in the times of pandemic, to our knowledge, there has been no evaluation of this tool in the follow-up of exclusively pediatric patients with COVID-19 after their care in an emergency service.

The studied series of pediatric cases reproduced previously published findings indicating that COVID-19 is a disease with less clinical severity in children than in adults,^14^ as evidenced by the lack of pediatric deaths related to the disease and the fact that most of the studied children became asymptomatic within 9 days after visiting the emergency department.

The main benefits resulting from the use of telemonitoring in this context are associated with (1) maintenance of social isolation, by reducing unnecessary visits to hospitals or clinics; (2) reduction of the time in the emergency department, enabling less exposure to and contact with other patients; and (3) increased safety, involving remote postdischarge follow-up by the emergency department with the possibility of early detection of clinical deterioration.

The most relevant difficulties associated with telemonitoring were related to the technological aspect of the service, such as possible connection failures, contact difficulties, or scheduling with families. Moreover, the relative insecurity of patients and their families about this type of remote health care provision presented a challenge in the development of the patient–emergency doctor relationship.

Among children who returned to an emergency service for a new in-person evaluation, all patients who returned under the indication of telemedicine were hospitalized, whereas only 2 of the 11 children who spontaneously returned were eventually judged to have an indication for hospitalization. This shows the clinical utility of telemonitoring in reducing unnecessary visits to emergency departments.^15^

The average length of hospitalization in the children who were subsequently admitted was shorter than that of the children admitted after the first in-person evaluation, which indicates that telemonitoring is safe and can detect the appropriate time of re-evaluation, without resulting in worsening of the patients' condition. Among the children with comorbidities, only one needed hospitalization, which proves that telemonitoring is also useful and applicable for children with previous diseases or comorbidities.

One of the strengths of this study is that telemedicine was investigated in an exclusively pediatric hospital in the city of São Paulo, Brazil, at a time when the number of COVID-19 cases was rapidly increasing^16^ and information about COVID-19 in children was still rare and uncertain.

However, the small number of patients in this study compromises the accuracy of the presented information. In addition, it is important to recognize that the study was conducted in a pediatric population from a private hospital in a large urban center in Brazil. In reality, access to telemedicine is still limited for the low-income population, owing to the lack of equity in internet access and the limited coverage by health operators for this new service. These issues still pose a challenge to the mass dissemination of telemedicine and to obtaining greater knowledge about the use of such technology.

The findings of this study corroborate the conclusion of other studies that sought to investigate the use of the technology in the health field: telemedicine is a useful resource in the care of patients with COVID-19.^15^

Further studies with a larger number of patients are needed to confirm our research data and to further prove the effectiveness of telemonitoring. New studies may also focus on the incorporation of telemedicine in hospital departments other than the emergency department and on the applicability of telemedicine in the monitoring of diseases other than COVID-19.

In conclusion, telemonitoring proved to be a useful resource for the continuity of care and identification of cases requiring hospitalization after visiting the emergency department of Hospital Sabará. Telemedicine is an effective and safe alternative for monitoring children diagnosed with COVID-19.

## Abbreviations Used

COVID-19: coronavirus disease 2019
OR: odds ratio
RT-PCR: reverse transcription polymerase chain reaction
SARS-CoV-2: severe acute respiratory syndrome coronavirus 2
